# A clustering approach to identify rare variants associated with hypertension

**DOI:** 10.1186/s12919-016-0022-0

**Published:** 2016-10-18

**Authors:** Rui Sun, Qiao Deng, Inchi Hu, Benny Chung-Ying Zee, Maggie Haitian Wang

**Affiliations:** 1 Division of Biostatistics, School of Public Health and Primary Care, Chinese University of Hong Kong, Shatin, Hong Kong; 2 Department of ISOM, Hong Kong University of Science and Technology, Clearwater Bay, Hong Kong

## Abstract

With the development of the next-generation sequencing technology, the influence of rare variants on complex disease has gathered increasing attention. In this paper, we propose a clustering-based approach, the clustering sum test, to test the effects of rare variants association by using the simulated data provided by the Genetic Analysis Workshop 19 with an unbalanced case-control ratio. The control individuals are (a) clustered into several subgroups, (b) statistics of the separate subcontrol groups as compared to the case group are calculated, and (c) a combined statistic value is obtained based on a distance score. Collapsing of rare variants is used together with the proposed method. In our results, comparing the same statistical test with and without clustering, the clustering strategy increases the number of true positives identified in the top 100 markers by 17.24 %. Compared to the sequence kernel association test, the proposed method is more robust in terms of replicated frequencies in the replicates data sets. The results suggest that the clustering approach could improve the power of nonparametric tests and that the clustering sum test has the potential to serve as a practical tool when dealing with rare variants with unbalanced case-control data in genome-wide case-control studies.

## Background

Genome-wide association studies have successfully detected a number of variants associated with complex traits and provided valuable insights into the genetic etiology of complex traits, but only a small portion of the total heritability has been explained [1]. This current situation leads to a question of the mysterious “missing heritability.” One possible source of missing heritability is the influence of rare variants under the common disease rare variant hypothesis [2, 3]. With the development of next-generation sequencing technology the whole genome can be sequenced, which makes the analysis of rare variants possible. Previously proposed methods to unveil associations of rare variants include the weighted sum statistic [4], combined multivariate and collapsing method [5], and the cohort allelic sums test [6]. In this study, we proposed a novel approach, namely, a clustering sum test (CST), to detect rare mutations. Specifically, the CST enables additional use of an individual’s quantitative phenotype information. One of the most important advantages of CST is the improvement in power to detect the effects of rare causal variants, comparing them to the original statistics without using additional parameters. We apply the proposed method to the simulated data set provided by the Genetic Analysis Workshop 19 (GAW19). The optimal collapsing window size is evaluated and the best result is compared to the sequence kernel association test (SKAT).

## Methods

### Data set

The GAW19 data set consists of the real whole genome sequencing genotype data and 200 replicates of simulated phenotypes, including the continuous systolic (SBP) and diastolic blood pressure (DBP) and hypertension status. In this study, we use the genotypes on chromosome 3, which include 48,510 single-nucleotide polymorphisms (SNPs). Quality control is conducted and SNPs are excluded if the percentage of missing value is more than 5 %, the minor allele frequency (MAF) equals zero, or an inconsistent genotype format exists. There are 1943 independent individuals and 42,825 SNPs that pass the quality control assessment with a MAF of less than 1 %.

### Association test for rare variants

Suppose a marker G has 3 genotypes *AA*, *Aa*, *aa* coded as 0, 1, 2. Where *a* refers to the minor allele. We want to analyze the association of G with the binary phenotype *Y*, as well as with its continuous phenotype information. In this study, a χ^2^ test is used to measure this association.

### Clustering and combination strategy

- Step 1. K-means clustering for individuals’ classification

In this step, a K-means clustering method [7] is used to cluster the control data into *K* groups, where *K* is an optimal group number determined by cross-validation [8]. Thus the *K* sets of control data have different average levels of hypertension.

- Step 2. Clustering sum test

Each control data set after clustering is matched with the same case data set. The χ^2^ test statistic is then calculated. The clustering and the χ^2^ test statistic are then combined with the weighted sum test.

The form of the clustering weighted sum test is:

1 $$ CST={\displaystyle {\sum}_{i=1}^K\frac{d_i}{D}{S}_i} $$

Where, *d*_*k*_ is the average phenotype distance between the *k* ^*th*^ control group and the case group. *D* is the sum of *d*_*k*_ and *S*_*k*_ is the χ^2^ test applied on the *k* ^*th*^ control and case data sets.

### Collapsing strategy

For all rare variants, SNPs within window size *L* are collapsed as pseudo markers. Window sizes *L* are chosen as 1, 10, 15, 20, 25, 30, 50, where one refers to no collapsing. CST is then applied to these pseudo markers and the optimal collapsing window size is chosen.

## Results

### Selection of optimal collapsing window size

Rare variants on chromosome 3 are collapsed using different window sizes and the statistical test is conducted to detect causal variants. A “pseudo marker” is 1 collapsed marker and a corresponding “pseudo answer” is the pseudo marker that contains at least 1 functional causal variant. Table 1 shows the mean number of true positives in top *n* pseudo markers in 50 replicates by using 3 methods, namely, the CST based on the χ^2^ test, the χ^2^ test, and SKAT [9], where *n* is set as 50, 100, 200, 300, 500, 750, 1000. Under the setting of chosen window size 15, CST-χ^2^ outperforms the χ^2^ test and SKAT in different selection criteria settings. This is especially true when the selection criteria is to be within the top 100 and 300; in those cases, the number of true positives identified is 44.7 and 35.9 % higher, respectively, than the numbers identified by SKAT.

**Table 1:** The number of true positives identified under collapsing window size 15

Top markers	CST-χ^2^	χ^2^ Test	SKAT
50	1.4	1.24	1.08
100	2.72	2.32	1.88
200	4.74	4.2	3.62
300	7.04	5.78	5.18
500	10.48	9.68	9.02
750	14.48	13.92	13.46
1000	18.62	18.74	17.6

**Fig. 1:**
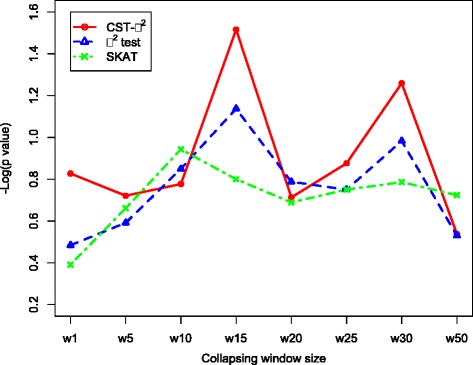
Compare *p* values for the number of pseudo answers identified at different collapsing window sizes in the top 100 pseudo markers by using Manhattan plot. Largest − Log(*p* value) indicates the best collapsing window. Optimal window sizes are pointed with *arrows*

The *p* value of each table count is estimated based on a binomial distribution to describe the probability of obtaining more than the observed number of answers under the null hypothesis of no associations. Window size 15 has been demonstrated to be the most significant window size when the criteria setting is top 100 markers (Fig. 1). When the same window size selection strategy is applied to the χ^2^ test and SKAT, the optimal window sizes are 15 and 10, respectively.

### Comparison of the effectiveness of clustering versus non-clustering

In this study, because CST is based on an χ^2^ test, the main difference between CST and the χ^2^ test is the clustering information. We use the results obtained using optimal window size 15 to compare the performance of the CST approach to the χ^2^ test. The numbers of pseudo answers identified in the top 100 and 300 markers are 2.72 and 7.04 using CST and 2.32 and 5.78using the χ^2^ test. In addition, we compare the general performance across different window sizes, that is, sizes other than 15. The significant levels of CST are still stronger than the χ^2^ test in most cases (see Fig. 1). In this respect, CST has a better performance and can identify more causal markers than can the χ^2^ test. This result indicates that involving more continuous phenotype information by clustering could increase the power of nonparametric tests.

### Comparison of clustering sum test to sequence kernel association test

In this part, we compare the number of causal markers identified by CST and SKAT under their optimal window sizes. Table 2 summarizes the information of the top 5 answers that CST and SKAT identified and provides several interesting findings.

**Table 2:** Top 5 pseudo answers identified by CST and SKAT

Method	Rank	Rep freq^a^	Gene	Number of SNPs identified	SNP with the strongest effect			Cumulative effect	
					Position	SBP	DBP	SBP	DBP
CST	1	22	*MAP4*	7	47912407	−20.621	−9.595	−114.19	−53.134
	2	16	*ZBTB38*	1	141164276	−0.007	−0.002	−0.007	−0.002
	3	9	*SEMA3F*	4	50225153	1.418	1.013	3.063	2.189
	4	7	*MLH1*	6	37092025	0	−0.449	0	−2.084
	5	7	*SEMA3F*	3	50222879	1.361	0.973	3.472	2.482
SKAT	1	10	*ZBTB38*	1	141164276	−0.007	−0.002	−0.007	−0.002
	2	6	*ARHGEF3*	2	56835799	−0.067	−0.062	−0.127	−0.117
	3	5	*MAP4*	1	48040284	−20.808	−9.682	−20.808	−9.682
	4	5	*FLNB*	1	58134409	1.687	0.249	1.687	0.249
	5	4	*MUC13*	1	124646631	0	−2.178	0	−2.178

^**a**^Replication frequency, replication times of being identified in 50 replicates

### Power and robustness of clustering sum test

In the top 2 pseudo answers detected, the replication frequencies of CST are as high as 22 and 16 out of the total 50 replicates, whereas they are 10 and 6 using SKAT. In the top 5 pseudo answers, the replication frequency is 96.3 % higher than it is for SKAT. These results show that CST is a more robust approach than SKAT for identifying rare variants. This may be because of the nonparametric nature of the CST, which could weaken the influence of noise from different types of underlying genetic architecture.

### Validation and effect size of answers identified

Information on causal markers for hypertension in the simulated data is provided by GAW19. The gene *MAP4* shows the strongest effect among causal genes in chromosome 3. Both the CST and the SKAT could detect *MAP4* in the top 5 markers, although the detected SNPs in *MAP4* are not the same. *MAP4* ranks first by the CST, and this pseudo marker includes seven answer SNPs with cumulative effects of −114.19 and −53.134 in SBP and DBP, respectively. Cumulative effects in *MAP4* identified by SKAT are −18.180 and −8.459 in SBP and DBP. The cumulative effect is calculated by the sum of SBP and DBP effects within the same collapsing window. In addition, CST identifies the pseudo marker containing more than three causal SNPs, whereas in the SKAT result, 4 of the top 5 pseudo answers contain only 1 causal SNP. From this perspective, CST can detect causal markers with the largest cumulative effect size in a robust manner whereas SKAT tends to detect markers containing a single SNP with a different effect size rather than with a large cumulative effect.

### Little-overlapping markers identified

Surprisingly, the findings by the two methods show very little overlap: only one marker is detected by both methods at SNP position 141164276 in gene *ZBTB38*. One gene, *MAP4*, could be detected in different regions by both methods. Findings of these two genes could further validate the effectiveness and accuracy of CST: the top marker identified is supported to have the strongest effect on hypertension and the second marker is the only overlapped variant identified and ranked as the topmost by SKAT. The small-overlapping pattern might be a result of the difference between the theoretically based hypothesis of the definition to measure the causal relationship between markers and disease of interest. One assumption behind collapsing is that the genetic probability distribution is similar within the same collapsing region. CST could match the assumption to enlarge the collapsed signals in the same effect direction. For SKAT, it is a kernel regression to detect the effect in each region and an inverse weight score is given to rare SNPs. SKAT is more useful and sensitive for detecting SNPs in a region with an opposite effect direction.

## Discussion

### Features of clustering sum test

In this paper, we propose a clustering-based test to detect rare variants when continuous phenotypes are available. There are three advantages of doing this: First, the CST can make better use of phenotype information, instead of just dichotomizing continuous phenotypes in a single case-control study. Second, when the ratio of number of cases to controls is extreme, the CST can balance the number of samples in each case-control set by dividing the larger group into smaller groups. Third, CST produces more robust results than the other tests because of its nonparametric form. All of the above findings suggest that CST has the potential to become a useful method in dealing with rare variants in case-control studies and thus is worth further investigation.

### Issue in terms of genetic architecture

The CST has been shown to outperform SKAT with larger power when the collapsing region contains many causal SNPs with the same effect direction. With the collapsing strategy applied, the CST, as a burden test, combines signals of rare variants within a certain region, thus, signals of cumulative effects could be enlarged for detecting whether the effects of causal variants are in the same direction. SKAT is based on a multiple regression to directly measure the relationship between phenotypes and multiple genetic variants in a region [9] and it is able to capture effects in a region with different directions. SKAT tends to detect a rare variant region with a single or a small number of causal markers with large effect. This phenomenon also suggests that extending the scope of knowledge in different genetic architectures could help when choosing an appropriate method in genetic association studies.

## Conclusions

Previous study on a similar version of GAW19 data sets indicates that the false-positive rate is usually high, as our results show in Table 1 [10]. The proposed CST approach has an attractive feature in that it increases power by using continuous phenotypes combined with categorical data tests for dichotomous phenotypes. It could be generalized to apply to multiple phenotypes by using different statistics to deal with unbalanced case-control data for genetic association tests.
